# Profiles in Nonverbal Learning Disability, Academic Skills, and Psychiatric Diagnoses in Children

**DOI:** 10.1001/jamanetworkopen.2025.33848

**Published:** 2025-10-01

**Authors:** Amy E. Margolis, Jacob DeRosa, Minji Kang, Prudence W. Fisher, Lauren Thomas, Caroline Southwick, Jessica Broitman, John M. Davis, Aki Nikolaidis, Michael P. Milham

**Affiliations:** 1 Department of Psychiatry and Behavioral Health, Wexner Medical Center, The Ohio State University, Columbus; 2 Child Mind Institute, New York, New York; 3 Department of Psychiatry, Columbia University Irving Medical Center, New York, New York; 4 Brooklyn Learning Center, Brooklyn, New York; 5 International Control Mastery Therapy Center, Berkeley, California; 6 California State University, East Bay; 7 Center for Biomedical Imaging and Neuromodulation, Nathan S. Kline Institute for Psychiatric Research, Orangeburg, New York

## Abstract

**Importance:**

Nonverbal learning disability (NVLD) has been described since the 1960s, with varying subtypes proposed to address clinical heterogeneity.

**Objective:**

To identify profiles of NVLD to improve clinical practice and increase rigor in research by parsing clinical heterogeneity.

**Design, Setting, and Participants:**

This cross-sectional study used data from the Healthy Brain Network (HBN; release 8, years 2017 and 2021), a study of brain development and youth mental health that enrolls a community sample of New York, New York, children and adolescents (ages 5-21 years), to identify youths who met the research-defined criteria for NVLD. HBN recruits for desire to participate in research or perceived clinical concern, yielding a high proportion of youths with behavioral, learning, or emotional problems. An unsupervised clustering approach, Louvain community detection, was applied to the diagnostic parameters that defined NVLD. Profiles were described by patterns of strengths and weaknesses across diagnostic tests and by associations with clinical symptoms. Data were analyzed between April 22 and September 27, 2021.

**Exposure:**

Perceived clinical concern.

**Main Outcomes and Measures:**

The outcome of interest was NVLD profiles; hypotheses were generated prior to data analysis.

**Results:**

Among 1640 children and adolescents with complete data in the HBN, 180 participants (110 [61%] male; 86 participants [48%] age 10-14 years; range, 6-17 years) met the research criteria for NVLD. Four profiles emerged: profile 1 (44 children) included deficits in both dimensions of visual-spatial processing (mean [SD] Wechsler Visual-Spatial Index [VSI], 96.11 [11.91]; Fluid Reasoning Index [FRI], 77.18 [7.94]), highest inattention (mean [SD], 1.23 [1.05]]; *P* = .001) and aggression scores (mean [SD], 65.03 [15.89]; *P* = .001), and lowest reading comprehension scores (mean [SD], 93.81 [10.31]; *P* = .001); profile 2 (37 children) included deficits in VSI (mean [SD], 78.27 [13.55]) but not FRI, the highest math scores (mean [SD], 101.16 [17.57]; *P* = .001) and rate of anxiety disorder (odds ratio, 2.19; 95% CI, 1.31-3.66; *P* = .02), and the lowest rate of specific learning disorder (odds ratio, 0.20; 95% CI, 0.05-0.84; *P* = .01); profile 3 (35 children) included deficits in FRI (mean [SD], 88.6 [12.63]) and highest reading comprehension scores (mean [SD], 101.8 [14.12]; *P* < .001); profile 4 had no deficits in VSI or FRI, lowest verbal intelligence (mean [SD], 87.12 [13.07]; *P* = .001), and no functional impairments. In profiles with visual-spatial deficits (profiles 1, 2, and 3), VSI and FRI scores were positively associated with scores on measures of functional impairment, eg, FRI and math in profile 2 (Pearson *r* = .33; *P* < .001).

**Conclusions and Relevance:**

In this cross-sectional study of heterogeneity in NVLD, 3 profiles reflected NVLD and were differentially associated with psychiatric and academic outcomes. One profile (profile 4) showed no specific visual-spatial deficits and no associations between visual-spatial processing and functional domains, pointing to a need to consider a revision to the NVLD research criteria.

## Key Points

**Question:**

Is there clinical heterogeneity in nonverbal learning disability (NVLD)?

**Findings:**

In this cross-sectional study including 180 children, 3 NVLD profiles emerged: (1) broad visual-spatial deficits with inattention, aggression, and poor reading comprehension; (2) isolated deficits in visual-spatial index, no math problems, high anxiety, and low specific learning disorder rates; and (3) deficits in Fluid Reasoning Index, math problems, and good reading skills. A fourth profile was characterized by broad, nonspecific weaknesses, spanning verbal and visual-spatial domains.

**Meaning:**

These findings suggest that NVLD has significant clinical heterogeneity and some children with broader deficits extending beyond the visual-spatial domain may merit reconsideration for a diagnosis; understanding the patterns of strengths and weaknesses can improve treatment research.

## Teaser

This cross-sectional study identifies and describes profiles of nonverbal learning disability through a Louvain community detection by diagnostic tests and by associations with clinical symptoms.

## Introduction

First described in the 1960s,^1^ nonverbal learning disability (NVLD) distinguishes children with deficits in visual-spatial reasoning from those with language-based (verbal) problems. Definitions of NVLD vary, with debate over whether core impairments lie solely in visual-spatial reasoning or also include deficits in social perception, motor skills, and executive function (EF).^2^ Two recent reviews noted variability in definitions but highlighted visual-spatial difficulties as central.^2,3^ Neuroimaging studies have used criteria focused on visual-spatial deficits,^4,5,6,7^ identifying disruptions in related brain circuits.^8^ In 2025, a working group proposed updated diagnostic criteria focused on these deficits, renaming the condition *developmental visual-spatial disorder* (DVSD).^9^

Acknowledging heterogeneity in NVLD, some researchers have proposed 2 to 4 subtypes characterized by differences, such as math vs social problems, or the presence of processing speed deficits.^10,11,12,13^ These studies used small samples and varying definitions, so NVLD heterogeneity remains unclear. Defining distinct neuropsychological profiles is vital for identifying less typical presentations and informing treatment. Subtypes may show unique strengths and vulnerabilities, including differing risks for common comorbidities, like anxiety or attention issues,^2,14^ requiring tailored interventions.

Drawing on prior studies,^4,5,6,7,8,15,16^ we developed a 4-part algorithm to define NVLD: (1) visual-spatial reasoning deficits, (2) impairment in at least 2 of 4 functional domains (EF, math, fine-motor, and social function), (3) intact word reading, and (4) absence of autism spectrum disorder (ASD) symptoms. This generates up to 11 NVLD profiles, potentially reflecting clinical subtypes. However, their prevalence, neuropsychological differences, and associated academic and psychiatric problems have not been empirically tested.

We used data from 180 children from the Healthy Brain Network (HBN)^17^ who met NVLD criteria. We applied Louvain community detection (LCD) to identify profiles based on our algorithm’s parameters. We hypothesized that LCD would reveal neuropsychological profiles similar to those in prior clinical literature (eg, math vs social problems^13^). We expected profiles to differ in academic and social impairment and in the nature of observed deficits, supporting NVLD’s heterogeneity. We describe the profiles and then test their associations with academic skills not included in our criteria (eg, reading comprehension), psychiatric diagnoses, and symptom counts. Finally, we explore links between visual-spatial abilities and functional impairment across and within profiles.

## Methods

This cross-sectional study was deemed exempt from review by the New York State Psychiatric Institute institutional review board because as a deidentified, secondary data analysis, it was not human participants research. The HBN was approved by the Advarra institutional review board. Before conducting research, written informed consent was obtained from participants 18 years or older; written informed consent from legal guardians and written informed assent were obtained from participants younger than 18 years.

### Participants

A total of 1640 participants in HBN (release 8) with complete data for the NVLD parameters were included. HBN is a study of brain development and youth mental health, enrolling children and adolescents (ages 5-21 years) who reside in New York, New York. HBN recruits on the basis of perceived clinical concern (mental health or learning), promoting the inclusion of a high proportion of youths with behavioral, learning, or emotional problems. Demographic data, including race and ethnicity, are self-reported. Race and ethnicity were self-reported by a parent or guardian using the following categories: Asian or Pacific Islander, Hispanic, non-Hispanic Black, non-Hispanic White, and other or 2 or more races or ethnicities. These variables were assessed to describe sample diversity and to evaluate potential differences in profiles across sociodemographic groups.

As described elsewhere,^17^ all neuropsychological assessment and clinical measures in HBN are administered by, or directly under the supervision of, licensed clinicians. Altogether, 180 youth met research criteria for NVLD and were included in this study.

### Measures Used to Assess Inclusion Criteria for NVLD

Research criteria for NVLD were met based on review of HBN data. We applied an algorithm for NVLD based on definitions used in prior studies^16,18^ with 4 inclusion criteria. First, visual-spatial deficit was based on performance on the Wechsler Intelligence Scale for Children–Fifth edition (WISC-V; range, 40-160; higher score indicates better performance),^19^ quantified by performance at or below the 16th percentile on the Block Design or on the Matrix Reasoning subtests, or a discrepancy of at least 15 standard score points higher on the Verbal Comprehension Index (VCI; range, 45-155; higher score indicates better performance) than on either the Fluid Reasoning Index (FRI; range, 45-155; higher score indicates better performance) or the Visual-Spatial Index (VSI; range, 45-155; higher score indicates better performance). Second, participants must have deficits in at least 2 of 4 functional domains based on prior studies: math (Wechsler Individual Achievement Test, third edition [WIAT-III] Numerical Operations; range, 40-160; higher score indicates better performance; deficit defined as ≤16th percentile),^20^ fine-motor (assessed using the Grooved Pegboard test; assessed as *z*-scores; higher score indicates better performance; deficit defined as ≤16th percentile),^21^ social (Child Behavior Checklist Social Problems; range, 0-100; higher score indicates more problems; deficit defined as T-score ≥70),^22^ or EF (specifically both inhibitory control [IC], measured with the National Institutes of Health [NIH] Toolbox: Flanker Inhibition Control and Attention,^23^ with higher score indicating better performance, and cognitive flexibility [CF], measured using the NIH Toolbox Dimensional Card Sort,^23^ with higher score indicating better performance]; deficit defined as ≤16th percentile). Third, we used proficient single-word reading to determine intact word reading as in prior studies (as measured by WIAT-III^20^ Word Reading subtest; range, 40-160; higher score indicates better performance; deficit defined as ≤16th percentile). Participants were excluded if they had a score of 20 or higher on the Autism Spectrum Screening Questionnaire.^24^ We note that 32 youth received an ASD diagnosis but did not have high screening scores for ASD.

### Measures Used to Assess Psychiatric and Academic Symptoms

Best-estimate psychiatric diagnoses and diagnostic consensus were achieved via case conference with interviewers and senior clinicians, including neuropsychology and psychiatry experts. All measure in HBN were used to determine diagnoses^17^; we describe only the measures used in the current analyses. Psychiatric symptoms were assessed via the Kiddie Schedule of Affective Disorders^25^ administered by trained interviewers to parent and youth; Screen for Child Anxiety Related Disorders (including generalized anxiety [range, 0-18], separation anxiety [range, 0-10], social anxiety [range, 0-16], panic disorder/somatic symptoms [range, 0-14], and school avoidance [range, 0-6]; higher score indicates greater anxiety)^26^ administered to parent and youth; Strengths and Weaknesses of Attnetion Deficit/Hyperactivity Disorder (ADHD) Symptoms and Normal Behavior Scale (range, −54 to 54; higher score indicates greater levels of ADHD symptoms)^27^ administered to parents, and the Conners ADHD Rating Scales Self-Report Short Form (T scores with a mean [SD] of 50 [10]; higher score indicates greater levels of ADHD symptoms).^28^ General impairment was assessed via the Columbia Impairment Scale (range, 0-52; higher score indicates greater impairment) administered to parent and youth.^29^ Academic and cognitive skills that were not part of the diagnostic criteria but that have been described as areas of challenge for some youth with NVLD were assessed via WISC-V Processing Speed Index (range, 45-155; higher score indicates better performance),^19^ Comprehensive Test of Phonological Processing (standard scores with mean [SD] of 100 [15]; higher score indicates better performance),^30^ and WIAT second edition^31^ Reading Comprehension subtest (standard scores with mean [SD] of 100 [15]; higher score indicates better performance). A full description of each measure is provided in the eMethods in Supplement 1. These measures were not included in the clustering procedure.

### Statistical Analysis

All, and only, measures used to assess inclusion criteria for NVLD were used as parameters in the LCD clustering model (eMethods in Supplement 1).^32,33^ The nodes of the graph are individuals and similarities are based on multivariate neuropsychological data.^34^

To describe each profile, we identified patterns of visual-spatial processing deficits by examining IQ indices and discrepancies between indices. An absolute deficit was defined by performance at least 1 SD (15 points) below the population mean. A relative deficit was defined by a difference of at least 15 points between verbal and visual-spatial scores (eg, VCI vs FRI or VSI). To describe patterns of impairment in math, fine-motor, social, and EF associated with each profile, we describe at-risk indicators (defined as 1 SD from the mean: *z*-score = 1; *t* = 60; standard score = 85; scaled score = 7) and clinically significant indicators (1.5 SDs from the mean: *z*-score = 1.5; *t* = 65; standard score = 77.5; scaled score = 5.5).

We present the mean values for each clustering parameter for each profile; analysis of variance tested differences between profiles on clustering parameters and Bonferroni adjustment corrected for multiple comparisons across the 11 parameters; Tukey post hoc analyses examined mean differences between profiles. Differences between profiles on demographic factors were assessed via χ^2^ and Bonferroni adjustment corrected for multiple comparisons (6 tests).

To characterize and validate the profiles, the association of each profile with psychiatric diagnoses was assessed with logistic regression, again with Bonferroni adjustment (7 tests). Differences between profiles on continuous clinical symptom variables and rates of categorical *Diagnostic and Statistical Manual of Mental Disorders* (Fifth Edition) (*DSM-5*) diagnoses were assessed via analysis of variance or χ^2^, respectively, with Bonferroni adjustment for multiple comparisons applied familywise within a domain (eg, anxiety, attention). Tukey post hoc analyses examined mean differences between profiles, and post hoc *Z*-tests examined *DSM-5* diagnosis proportion of differences between profiles. To examine cooccurring diagnoses in the sample, χ^2^ tests of independence evaluated whether the frequency of *DSM-5*–based clinical diagnoses (eg, ADHD) varied across the LCD-derived subtypes (eMethods in Supplement 1). Pearson correlations evaluated associations between each index of visual-spatial ability (eg, FRI, VSI) and 1 of 4 areas of possible impairment (EF, math, fine-motor, and social function) within each profile and across all participants to determine if visual-spatial deficits might underlie these impairments.

*P* values were 2-sided, and statistical significance was set at *P* ≤ .05. Analyses were performed in R software version 4.1.2 (R Project for Statistical Computing) and Python software version 3.8.10 (Python Software Foundation), using igraph version 1.2.7 in R and python igraph version 0.9.6 for LCD, scikit-learn version 0.24.2 and statsmodels version 0.12.2 for modeling, and NumPy version 1.20.3, pandas version 1.3.5, and SciPy version 1.7.3 for data handling and statistical tests. Data were analyzed between April 22 and September 27, 2021.

## Results

### Participants

A total of 180 participants (110 [61%] male; 86 participants [48%] aged 10-14 years) met the research criteria for identifying NVLD, yielding a demographically diverse sample, ranging in age from 6 to 17 years; 8 participants (4%) identified as Asian or Pacific Islander, 40 participants (22%) as Hispanic, 44 participants (24%) as non-Hispanic Black, 73 (41%) as non-Hispanic White, and 15 participants (8%) as other or 2 or more races or ethnicities (Table 1). Most families (98 [55%]) reported income above $100 000, 42 (24%) reported incomes between $50 000 and $100 000, and 37 (21%) reported income less than $50 000.

**Table 1.** Sample Characteristics

Characteristic	Participants, No. (%)					χ^2^	*P* value
	Overall (N = 180)	Profile 1 (n = 44)	Profile 2 (n = 37)	Profile 3 (n = 35)	Profile 4 (n = 64)		
Sex							
Female	70 (39)	19 (43)	15 (41)	15 (43)	21 (33)	1.61	.66
Male	110 (61)	25 (57)	22 (59)	20 (57)	43 (67)		
Age, y							
6-9	65 (36)	15 (34)	12 (32)	14 (40)	24 (38)	8.64	.20
10-14	86 (48)	24 (55)	16 (43)	12 (34)	34 (53)		
15-17	29 (16)	5 (11)	9 (24)	9 (26)	6 (9)		
Race and ethnicity							
Asian or Pacific Islander	8 (4)	0	2 (5)	3 (9)	3 (5)	9.19	.69
Hispanic	40 (22)	11 (25)	8 (22)	8 (23)	13 (20)		
Non-Hispanic Black	44 (24)	12 (27)	9 (24)	4 (11)	19 (30)		
Non-Hispanic White	73 (41)	18 (41)	16 (43)	17 (49)	22 (34)		
Other/≥2 races	15 (8)	3 (7)	2 (5)	3 (9)	7 (11)		
Household income, $							
>50 000	37 (21)	6 (14)	9 (24)	5 (15)	17 (27)	6.85	.34
50 000-99 000	42 (24)	11 (26)	6 (16)	7 (21)	18 (29)		
≥100 000	98 (55)	26 (60)	22 (59)	22 (65)	28 (44)		
Parent 1 education							
<High school	10 (6)	3 (7)	3 (8)		4 (6)	11.81	.46
High school graduate	16 (9)	1 (2)	2 (5)	3 (9)	10 (16)		
Partial College	17 (10)	4 (9)	5 (14)	2 (6)	6 (9)		
College education	72 (40)	17 (40)	14 (38)	18 (53)	23 (36)		
Graduate degree	63 (35)	18 (42)	13 (35)	11 (32)	21 (33)		
Parent 2 education							
<High school	12 (8)	3 (9)	1 (3)	1 (3)	7 (14)	17.16	.14
High school graduate	31 (21)	11 (33)	5 (17)	3 (10)	12 (24)		
Partial College	26 (18)	4 (12)	6 (20)	9 (29)	7 (14)		
College education	41 (28)	6 (18)	12 (40)	12 (39)	11 (22)		
Graduate degree	35 (24)	9 (27)	6 (20)	6 (19)	14 (27)		

### Profile Characterization

**Figure 1.**
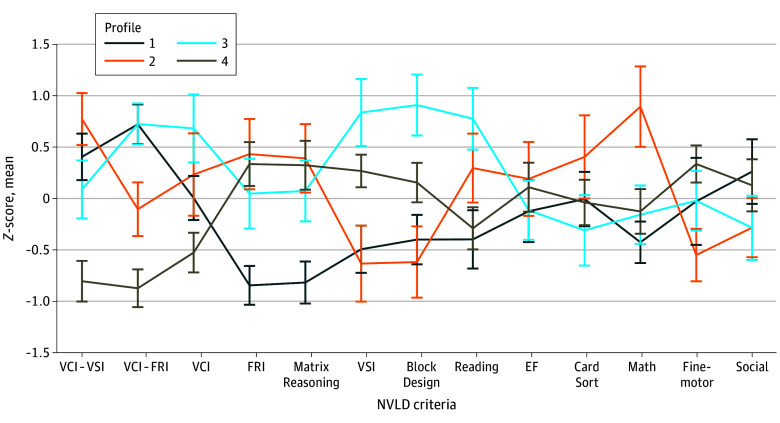
Nonverbal Learning Disability (NVLD) Louvain Community Detection Profiles Mean normalized profile loadings are on the y-axis as *z*-scores. NVLD criteria used in clustering analysis are presented along the x-axis. Block Design, Matrix Reasoning, Fluid Reasoning Index (FRI), Visual-Spatial Index (VSI), and Verbal Comprehension Index (VCI) were measured via the Wechsler Intelligence Scale for Children–Fifth edition^17^; fine-motor, Grooved Pegboard^19^; social, via Child Behavior Checklist Social Problems^20^; executive functioning (EF), NIH Toolox Flanker and Dimensional Card Sort^21^; and reading and math, Wechsler Individual Achievement Test-III Word Reading and Numerical Operations^18^ subtests.

Four profiles emerged (Figure 1; eFigure 1 and eFigure 2 in Supplement 1). Table 2 presents the number of children and the mean value for each parameter by profile. The 4 profiles did not differ in sex, age, race and ethnicity, household income, or parental education. Profile 1 had deficits in both FRI (mean [SD], 77.18 [7.94]; *P* = .001) and VSI (mean [SD], 96.11 [11.91]; *P* = .001) and the most areas of impairment. Profile 2 had a deficit in VSI (mean [SD], 78.27 [13.55]; *P* = .001) and the highest math skills (mean [SD], 101.16 [17.57]; *P* = .001). Profile 3 had a deficit in FRI (mean [SD], 88.6 [12.63]; *P* = .001). Profile 4 did not have deficits in VSI or FRI, had the lowest VCI scores (mean [SD], 87.12 [13.07]) and no impairments. Profiles did not differ on EF, with all but 1 profile in the at-risk range on 1 measure of EF. Profiles did not differ in social problems, with only 1 profile in the at-risk range.

**Table 2.** Nonverbal Learning Disability Parameters by Profile

Measure	Mean (SD)				*F*	*P* value	Post hoc^a^
	Profile 1 (n = 44)	Profile 2 (n = 37)	Profile 3 (n = 35)	Profile 4 (n = 64)			
VCI − VSI	16.14 (11.24)	21.7 (11.47)	11.34 (12.41)	−2.16 (11.97)	38.99	<.001	2 > 3 > 4 < 1
VCI − FRI	18.93 (9.61)	6.46 (11.84)	18.97 (8.78)	−5.16 (11.09)	62.43	<.001	4 < 1 < 2 < 3
VCI	96.11 (11.91)	99.97 (20.34)	107.57 (16.33)	87.12 (13.07)	14.82	<.001	3 > 1 > 4 < 2
FRI	77.18 (7.94)	93.51 (13.12)	88.6 (12.63)	92.28 (10.93)	20.02	<.001	1 < 2 = 3 = 4
VSI	79.98 (9.24)	78.27 (13.55)	96.23 (11.59)	89.28 (7.77)	25.66	<.001	3 > 4 > 1 = 2
Reading	97.23 (11.18)	105.54 (12.04)	111.29 (10.46)	98.52 (9.85)	15.1	<.001	2 = 3 > 4 = 1
EF	77.3 (11.3)	80.89 (12.34)	77.4 (9.57)	79.97 (10.92)	1.11	>.99	NA
Card Sort	82.89 (11.36)	88.24 (15.83)	78.91 (13.02)	82.45 (11.56)	3.31	.24	NA
Math	81.41 (9.95)	101.16 (17.57)	85.43 (12.4)	85.91 (13.04)	16.55	<.001	2 > 1 = 2 = 4
Fine-motor	−1.7 (3.32)	−2.94 (1.83)	−1.68 (2.02)	−0.83 (1.72)	6.69	.003	2 < 4
Social	62.52 (9.24)	57.65 (7.71)	57.63 (8.12)	61.33 (9.07)	3.5	.18	NA

Abbreviations: EF, executive functioning; FRI, Fluid Reasoning Index; NA, not applicable; VCI, Verbal Comprehension Index; VSI, Visual-Spatial Index.

^a^ Only significant post hoc tests are reported, combined for ease of reading, eg, 1 < 2 = 3 = 4 indicates that profile 1 was statistically significantly lower than profiles 2, 3, and 4, and there were no other differences between groups.

Profile 1 (44 participants) was characterized by VSI and FRI scores more than 1 SD below the population mean, discrepancies between VCI and VSI and between VCI and FRI, the lowest mean FRI (Table 2). Furthermore, participants in profile 1 were in the clinically significant range for inhibitory control and motor coordination problems and the at-risk range for social, cognitive flexibility, and math problems.

Profile 2 (37 participants) was characterized by VSI scores more than 1 SD below the population mean, the lowest VSI, a discrepancy between VCI and VSI, and the highest FRI and math scores (Table 2). Participants in profile 2 were in the clinically significant range for fine-motor problems and the at-risk range for inhibitory control problems (Table 2).

Profile 3 (35 participants) was characterized by a discrepancy between VCI and FRI and the highest VSI and VCI (Table 2). Participants in profile 3 were in the clinically significant range for inhibitory control and fine-motor coordination problems and the at-risk range for cognitive flexibility and math problems.

Profile 4 (64 participants) was characterized by Block Design scores 1 SD below the population mean (eTable 1 in Supplement 1), but neither VSI nor FRI scores were more than 1 SD below the mean, nor were there differences between VCI and either FRI or VSI. Nearly one-third of participants fit this profile. The profile is further characterized by the lowest mean VCI (Table 2). Participants in profile 4 were in the at-risk range for social, cognitive flexibility, and inhibitory control problems but showed no functional impairments in the clinically significant range.

### Profile Differences by Academic and Psychiatric Symptoms

**Figure 2.**
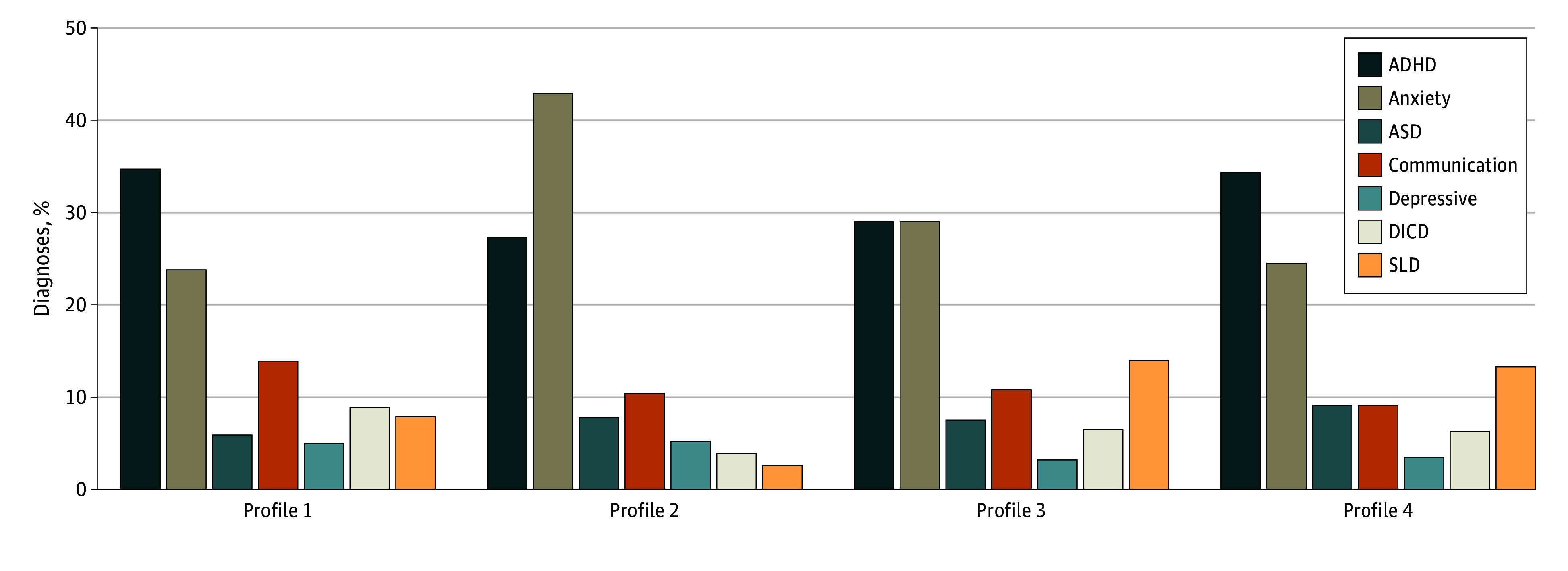
Rates of *Diagnostic and Statistical Manual of Mental Disorders* (*Fifth Edition*) Diagnoses by Profile Percentage of the individual diagnosis compared with the other diagnoses of interest by profile are shown on the y-axis. ADHD indicates attention-deficit/hyperactivity disorder; ASD, autism spectrum disorder; DICD, disruptive, impulse-control, and conduct disorders; SLD, specific learning disorder.

Profiles 2 and 3 had higher reading comprehension scores than profiles 1 and 4 (Table 3). Profiles did not differ by risk for or rates of ADHD (Figure 2; eTable 2, eTable 3, and eFigure 3 in Supplement 1). Profile 1 had significantly higher parent-reported symptoms of inattention than profile 2, and more self-reported aggression than profiles 2 and 3 (Table 3). Compared with other profiles, profile 2 had higher odds of anxiety disorder (OR, 2.19; 95% CI, 1.31 to 3.66; *P* = .02) (eTable 2 in Supplement 1) and lower odds of specific learning disorder (OR, 0.20; 95% CI, 0.05 to 0.84; *P* = .01) (Figure 2 and eTable 2 in Supplement 1). Across all participants, ADHD and anxiety had higher occurrences compared with all the other cooccurring diagnoses but did not differ from each other. There were no differences between profiles in risk for or rates of depressive disorders; disruptive, conduct, or impulse control disorders; communication disorders; or ASD (Figure 2; eTable 2, eTable 3, and eFigure 3 in Supplement 1).

**Table 3.** Psychiatric and Academic Symptoms Measures by Profile

Measure	Mean (SD)				F	*P* value	Post hoc^a^
	Profile 1 (n = 44)	Profile 2 (n = 37)	Profile 3 (n = 35)	Profile 4 (n = 64)			
Anxiety: SCARED							
Self report							
Generalized	6.37 (5.1)	4.62 (4.01)	6.26 (4.73)	5.87 (4.95)	0.98	>.99	NA
Panic	6.61 (6.21)	4.44 (4.97)	7.19 (6.18)	6.39 (6.3)	1.34	>.99	NA
Social	6 (4.61)	4.82 (3.46)	6.55 (3.85)	5.83 (4.21)	1.02	>.99	NA
Separation	2.42 (2.31)	1.47 (1.71)	2.61 (1.98)	2.43 (1.99)	2.26	.50	NA
School phobia or school avoidance	5.82 (4.27)	4.85 (3.2)	5.84 (4.5)	5.28 (4.59)	0.44	>.99	NA
Total	27.21 (18.88)	20.21 (13.67)	28.45 (17.42)	25.8 (19.09)	1.43	>.99	NA
Parent report							
Generalized	6.35 (4.53)	5.5 (4.9)	6.4 (5.34)	4.35 (3.96)	2.22	.52	NA
Panic	2 (3.3)	1.94 (2.46)	2.46 (3.3)	1.74 (3.04)	0.41	>.99	NA
Social	4.05 (3.74)	4.56 (3.73)	4.2 (3.31)	3.94 (3.86)	0.23	>.99	NA
Separation	1.49 (1.78)	1.17 (1.4)	1.69 (1.89)	1.24 (1.73)	0.75	>.99	NA
School phobia or school avoidance	3.77 (3.32)	2.94 (3.42)	3.51 (3.52)	2.87 (3.76)	0.7	>.99	NA
Total	17.65 (13.98)	16.11 (12.69)	18.26 (13.57)	14.23 (12.48)	0.92	>.99	NA
Attention: SWAN							
Inattention	1.23 (1.05)	0.5 (1.19)	1.01 (1.02)	0.91 (0.99)	3.41	.04	2 < 1
Hyperactivity	0.52 (1.02)	0.04 (1.3)	0.01 (1.17)	0.46 (1.12)	2.33	.15	NA
Behavioral symptoms and impairment							
CIS self-reported total	12.32 (10)	7.62 (8.42)	10.73 (7.87)	10.8 (9.74)	1.46	>.99	NA
CIS parent-reported score	13.38 (8.29)	9.38 (6.7)	13.09 (8.64)	12.24 (8.95)	1.61	>.99	NA
C3SR aggression T-score	65.03 (15.89)	53.03 (13.23)	54.48 (11.87)	59.59 (16.08)	4.94	.02	2 = 3 < 1
C3SR FR T	59.63 (15.56)	53.47 (11.13)	53.39 (12.8)	55.72 (13.41)	1.71	>.99	NA
C3SR hyperactivity T-score	67.03 (13.32)	58.47 (13.47)	60.03 (13.49)	62.09 (13.86)	2.72	.33	NA
C3SR inattention T-score	72.42 (14.03)	60.74 (15.01)	67.81 (14.88)	68.61 (15.51)	3.8	.08	NA
C3SR LP T	66.68 (13.14)	57.97 (11.96)	62.26 (12.95)	63.59 (12.4)	2.96	.24	NA
Processing speed							
WISC PSI	81.86 (12.93)	88.59 (18.52)	83.31 (14.16)	86.83 (12.45)	1.96	.49	NA
CTOPP RD	8.86 (2.69)	10 (2.48)	8.8 (2.69)	8.91 (2.78)	1.76	.63	NA
CTOPP RL	8.3 (2.55)	9.35 (2.52)	7.71 (2.63)	8.33 (2.71)	2.46	.26	NA
CTOPP RSN	92 (14.74)	98.81 (14.19)	90.11 (15.22)	92.2 (15.57)	2.39	.28	NA
WIAT RC	93.81 (10.31)	101.41 (13.6)	101.8 (14.12)	91.91 (12.55)	7.37	<.001	1 = 4 < 2 = 3

Abbreviations: C3SR, Conners 3 Self Report; CIS, Columbia Impairment Scale; CTOPP, Comprehensive Test of Phonological Processing; FR, family relations; LP, learning problems; NA, not applicable; PSI, Processing Speed Index; RC, reading comprehension; RD, rapid digit naming; RL, rapid letter naming; RSN, rapid symbolic naming; SCARED, Screen for Child Anxiety Related Disorders; SWAN, Strengths and Weaknesses of ADHD Symptoms and Normal Behavior Scale; VSI, Visual Spatial Index; WIAT, Wechsler Individual Achievement Test; WISC, Wechsler Intelligence Scale for Children.

^a^ Only significant post hoc tests are reported, combined for ease of reading, eg, 1 < 2 = 3 = 4 indicates that profile 1 was statistically significantly lower than profiles 2, 3, and 4, and there were no other differences between groups.

### Associations Between Visual-Spatial Reasoning and Domains of Functional Impairment

VSI and FRI were positively associated with EF (IC), math, fine-motor, and social skills for the sample as a whole (eFigure 4 in Supplement 1). For VSI, associations with math and social skills were highest in profiles 2 and 3, associations with EF were highest in profiles 3 (IC) and 4 (CF), and associations with fine-motor skills were highest in profiles 1 and 2. For FRI, associations with math, fine-motor, and social skills were highest in profiles 1, 2, and 3, and associations with EF were highest in profiles 2 (CF) and 3 (IC). Among domains of impairment, math was positively associated with EF, which was driven by associations with IC. Social skill was positively associated with IC and fine-motor skill across all participants; this association did not vary by profile.

## Discussion

In this cross-sectional study, we examined profiles of children and adolescents meeting research criteria for NVLD in a large transdiagnostic sample to explore clinical heterogeneity. LCD identified 4 profiles with distinct visual-spatial strengths and weaknesses. These profiles were linked to external measures of academic and psychiatric symptoms, indicating that the clustering captured meaningful variation. Visual-spatial capacities were associated with all 4 functional domains (EF, math, fine-motor, and social), supporting the idea that visual-spatial deficits, the proposed core feature of NVLD, may underpin broader impairments. Profile 1, marked by deficits across all visual-spatial areas (construction, motor integration, and inductive and quantitative reasoning), showed the most functional impairments, pointing to a severity model. Differences in visual-spatial and functional patterns across profiles suggest that specific visual-spatial deficits may lead to unique challenges. Finally, consistent with prior descriptions of heterogeneity in NVLD,^10,11,12,13^ profiles varied in academic and psychiatric problems.

We defined NVLD based on a core deficit in visual-spatial reasoning^4,16^ rather than a syndromic definition used in some prior studies.^2^ Using a data-driven approach, we identified a severity model: 1 profile showed the most visual-spatial deficits and the most functional impairments. Two other profiles showed specific deficits—either visual-spatial construction/visual-motor integration or inductive and quantitative reasoning—with distinct associated impairments. Supporting our visual-spatial definition, we found these abilities were associated with functional impairments within and across profiles, consistent with prior work connecting visual-spatial reasoning with social, executive, fine-motor, and math problems in both typical and clinical populations.^35,36,37,38,39,40,41^ Future research using more targeted measures of visual-spatial skills may help refine treatment strategies.

The LCD-derived profiles showed varying patterns of visual-spatial deficits, aligning in part with clinical and empirical descriptions.^11^ Profile 2 identified deficits in visual-spatial construction but not fluid reasoning, similar to clinical cases 2 and 3 in the vignettes described by Grodzinsky et al.^11^ Our findings further converge with the observation from Grodzinsky et al^11^ of an NVLD subgroup with elevated anxiety symptoms, although further detail on visual-spatial deficits in those subtypes is needed to clarify overlap. Consistent with work by Forrest,^13^ we identified a profile (profile 3) characterized by math but not social impairments. Fine-motor difficulties appeared in 3 of 4 profiles, supporting prior findings that motor problems are common in NVLD,^12^ although some studies do not report this.^2^ Overall, the profiles identified reflect distinct patterns of visual-spatial strengths and weaknesses and show partial convergence with previously proposed NVLD subtypes based on differing NVLD criteria.

Links between visual-spatial reasoning and math have long been noted in both typical youth and those with impairment in math.^37^ In our sample, math performance correlated more strongly with FRI than VSI. Math problems were only observed when FRI, not VSI, was deficient, suggesting that inductive and quantitative reasoning (indexed by FRI) may be essential for math skills in NVLD. This points to FRI as a possible target for remediation or accommodation. Regarding EF, profile 2 showed high FRI and math scores but impairment in inhibitory control, whereas profiles with low FRI and math had deficits in both IC and cognitive flexibility. These results suggest a link between cognitive flexibility, FRI, and math in NVLD, highlighting cognitive flexibility as another intervention target.

Our findings showing profile-specific comorbidity patterns support the theoretical view that NVLD is a discrete clinical entity and is not better accounted for by currently available diagnoses. Profile 2 showed elevated risk of anxiety disorder, suggesting that NVLD is not simply a proxy for anxiety disorder. Furthermore, anxiety disorder occurred most often among individuals who also showed deficits in EF and fine-motor skills, hallmark features of clinical affective disorders.^42,43^ Although ADHD diagnoses did not differ across profiles, profile 1 had the most parent-reported symptoms of inattention, aligning with prior NVLD subtypes.^11^ Profile 1 also had the highest level of self-reported aggression symptoms, diverging from most reports of internalizing rather than externalizing behaviors in NVLD.^2^ Thus, our findings point to NVLD as a distinct clinical disorder and the clinical importance of understanding heterogeneity within the disorder. Notably, 32 youths with ASD diagnoses remained after screening, but they were evenly distributed across profiles, suggesting no ASD-specific cluster and that NVLD is not merely an ASD subtype.

Our findings suggest NVLD research criteria may need revision. Profile 4, approximately one-third of the sample, showed only 1 visual-spatial deficit and no VCI vs VSI or VCI vs FRI differences, indicating that the algorithm may not reliably identify visual-spatial problems. Functional impairment was not associated with VSI or FRI in 9 of 10 analyses, suggesting that any psychiatric or academic challenges these individuals experience do not derive specifically from visual-spatial deficits. Finally, no clinically significant impairment was found across domains. These data suggest that this profile likely does not represent NVLD, pointing to a downward revision of prevalence estimates to 2% to 3% of individuals, given the percentage of youth in profile 4 relative to the entire sample.^16^ Future studies might require at least 2 deficient visual-spatial scores for diagnosis.

Recently, NVLD has been reconceptualized as DVSD,^9,44^ placing the disorder in the context of the psychiatric diagnostic nomenclature by describing it based on a core deficit in a single domain of function, eg, attention disorder or motor disorder, in this case visual-spatial reasoning ability. Importantly, this reconceptualization removes learning disability from the name of the disorder, distinguishing it from specific learning disorder. Our finding that risk of specific learning disorder varied by profile further supports this distinction and reconceptualization of NVLD as a distinct neurodevelopmental disorder that is not a subcategory of specific learning disorder. Our data suggest that NVLD serves as a strong example of a dimensional approach to neurodevelopmental disorders, similar to ADHD, as it appears to be primarily characterized by deficits in visuospatial processing, which may be accompanied by varying patterns of strengths and weaknesses across individuals, reflected in the identified subprofiles. Our definition of NVLD aligns with DVSD, suggesting that NVLD subtypes may generalize to youth with DVSD. Our finding that different aspects of visual-spatial reasoning are affected in different subtypes points to the need for more fine-grained assessment of youth visual-spatial reasoning. Future studies are needed to develop such measures of visual-spatial reasoning, either via test performance or behavioral report, and to further refine our understanding of neuropsychological profiles of DVSD.

### Limitations

Although our findings are important, our study has some limitations. First, the sample is not representative of the general population but instead is weighted for children with neurodevelopmental problems and highly educated parents. Second, a clinician did not make the NVLD diagnosis; rather, inclusion criteria for NVLD were evaluated through a review of scores on measures completed during a clinical assessment. Future studies should include clinical assessment of NVLD/DVSD measuring visual-spatial reasoning abilities via test performance and clinical interview and examine whether profiles vary by degree of comorbid disorders. Although associations between profiles and clinical symptoms suggest the profiles are clinically meaningful, future studies should test differential associations between visual-spatial deficits and clinical outcomes. Future work could adapt a mixtures analysis approach (eg, weighted quantile sum regression) to identify whether there are some symptoms that are more related to impairment than other symptoms among the cluster of symptoms needed to generate a provisional NVLD diagnosis. Furthermore, our algorithm identified 4 profiles, although up to 11 distinct subtypes (based on combinations of domains of impairment) could be generated by the research criteria. Given limitations of our sample size, we were unable to test for the presence of all 11 subtypes, but the 4 identified profiles likely represent overarching categories of visual-spatial deficits, given their clear cognitive boundaries.

## Conclusion

In this cross-sectional study using a data-driven clustering approach, we identified 3 NVLD profiles distinguished by psychiatric and academic symptoms, and 1 profile of youths who may merit reconsideration for a diagnosis. Profile 1 included deficits in all areas of visual spatial reasoning ability assessed and all functional domains, as well as problems with inattention, aggression, and reading comprehension. Profile 2 included deficits in VSI only, no math problems, and the highest rates of anxiety disorder but lowest rates of specific learning disorder. Profile 3 included deficits in FRI, math problems, and good reading comprehension. Across profiles, visual-spatial reasoning was linked with functional outcomes—math, fine-motor, social, and EF skills—highlighting the importance of assessing distinct dimensions of visual-spatial ability. Our findings support a dimensional approach to characterizing NVLD/DVSD and underscore the need for comprehensive, normed tools to improve assessment and evaluate treatment efficacy. Finally, our results suggest a possible revision to the NVLD criteria to better capture individual-level deficits, which may lead to lower prevalence estimates.

## Notes

Supplement 1.
eMethods.
**eTable 1.** Subtest Means by Profile **eTable 2.** *DSM-5* Diagnosis Odds Ratios by Profile **eTable 3.** *DSM-5* Diagnoses by Profile **eFigure 1.** Louvain Bagging Cluster Stability Proportions **eFigure 2.** Unique Profiles Obtained Across 1000 Bootstrapped Iterations **eFigure 3.** NVLD Comorbid Diagnosis Prevalence Differences. **eFigure 4.** Visual-Spatial Reasoning Ability and Domains of Functional Impairment by Louvain Clustering Profiles
eReferences.

Supplement 2.
Data Sharing Statement

## References

[1] Johnson DJ, Myklebust HR. Learning Disabilities; Educational Principles and Practices. Grune & Stratton; 1967.

[2] Fisher PW, Reyes-Portillo JA, Riddle MA, Litwin HD. Systematic Review: Nonverbal Learning Disability. J Am Acad Child Adolesc Psychiatry. 2022;61(2):159-186. 33892110 10.1016/j.jaac.2021.04.003

[3] Mammarella IC, Cornoldi C. An analysis of the criteria used to diagnose children with nonverbal learning disability (NLD). Child Neuropsychol. 2014;20(3):255-280. doi:10.1080/09297049.2013.796920 23705673

[4] Margolis AE, Pagliaccio D, Thomas L, Banker S, Marsh R. Salience network connectivity and social processing in children with nonverbal learning disability or autism spectrum disorder. Neuropsychology. 2019;33(1):135-143. doi:10.1037/neu0000494 30411904 PMC6322976

[5] Banker SM, Pagliaccio D, Ramphal B, Thomas L, Dranovsky A, Margolis AE. Altered structure and functional connectivity of the hippocampus are associated with social and mathematical difficulties in nonverbal learning disability. Hippocampus. 2021;31(1):79-88. doi:10.1002/hipo.23264 32949475 PMC7749072

[6] Ramphal B, Pagliaccio D, Thomas LV, He X, Margolis AE. Contributions of cerebellar white matter microstructure to social difficulty in nonverbal learning disability. Cerebellum. 2021;20(6):931-937. doi:10.1007/s12311-021-01265-4 33856654 PMC8530438

[7] Semrud-Clikeman M, Fine JG, Bledsoe J, Zhu DC. Magnetic resonance imaging volumetric findings in children with Asperger syndrome, nonverbal learning disability, or healthy controls. J Clin Exp Neuropsychol. 2013;35(5):540-550. doi:10.1080/13803395.2013.795528 23672532

[8] Banker SM, Ramphal B, Pagliaccio D, . Spatial network connectivity and spatial reasoning ability in children with nonverbal learning disability. Sci Rep. 2020;10(1):561. doi:10.1038/s41598-019-56003-y 31953441 PMC6969178

[9] Fisher PW, Litwin HD, Riddle MA, Margolis AE. Report of a work group on nonverbal learning disability: consensus criteria for developmental visual-spatial disorder: reconceptualizing nonverbal learning disability for *DSM* consideration. J Am Acad Child Adolesc Psychiatry. 2025;64(8):882-896. doi:10.1016/j.jaac.2025.01.007 39828037

[10] Davis JM, Broitman J. NVLD and subtypes. In: Davis JM, Broitman J, eds. Nonverbal Learning Disabilities in Children: Bridging the Gap Between Science and Practice. Springer New York; 2011:13-19. doi:10.1007/978-1-4419-8213-1_3

[11] Grodzinsky GM, Forbes PW, Bernstein JH. A practice-based approach to group identification in nonverbal learning disorders. Child Neuropsychol. 2010;16(5):433-460. doi:10.1080/09297041003631444 20589542

[12] Ris MD, Ammerman RT, Waller N, . Taxonicity of nonverbal learning disabilities in spina bifida. J Int Neuropsychol Soc. 2007;13(1):50-58. doi:10.1017/S1355617707070087 17166303

[13] Forrest BJ. The utility of math difficulties, internalized psychopathology, and visual-spatial deficits to identify children with the nonverbal learning disability syndrome: evidence for a visualspatial disability. Child Neuropsychol. 2004;10(2):129-146. doi:10.1080/09297040490911131 15590491

[14] Fine JG, Semrud-Clikeman M, Bledsoe JC, Musielak KA. A critical review of the literature on NLD as a developmental disorder. Child Neuropsychol. 2013;19(2):190-223. doi:10.1080/09297049.2011.648923 22385012

[15] Semrud-Clikeman M, Fine JG, Bledsoe J. Comparison among children with children with autism spectrum disorder, nonverbal learning disorder and typically developing children on measures of executive functioning. J Autism Dev Disord. 2014;44(2):331-342. doi:10.1007/s10803-013-1871-2 23812759

[16] Margolis AE, Broitman J, Davis JM, . Estimated Prevalence of nonverbal learning disability among North American children and adolescents. JAMA Netw Open. 2020;3(4):e202551. doi:10.1001/jamanetworkopen.2020.2551 32275324 PMC7148441

[17] Alexander LM, Escalera J, Ai L, . An open resource for transdiagnostic research in pediatric mental health and learning disorders. Sci Data. 2017;4(1):170181. doi:10.1038/sdata.2017.181 29257126 PMC5735921

[18] Semrud-Clikeman M, Walkowiak J, Wilkinson A, Minne EP. Direct and indirect measures of social perception, behavior, and emotional functioning in children with Asperger’s disorder, nonverbal learning disability, or ADHD. J Abnorm Child Psychol. 2010;38(4):509-519. doi:10.1007/s10802-009-9380-7 20084452

[19] Wechsler D. Wechsler Intelligence Scale for Children: Technical and Interpretive Manual. 5th ed. Pearson; 2014.

[20] Wechsler D. Wechsler Individual Achievement Test: WIAT III. 3rd ed. Pearson; 2009.

[21] Strauss E, Sherman E, Spreen O. A Compendium of Neuropsychological Texts: Administration, Norms, and Commentary. Oxford University Press; 2006.

[22] ASEBA. School-age: (CBCL, TRF, YSR, BPM/6-18). Accessed August 3, 2021. https://aseba.org/school-age/

[23] Zelazo PD, Anderson JE, Richler J, Wallner-Allen K, Beaumont JL, Weintraub S. II: NIH Toolbox Cognition Battery (CB): measuring executive function and attention. Monogr Soc Res Child Dev. 2013;78(4):16-33. doi:10.1111/mono.12032 23952200

[24] Kopp S, Gillberg C. The Autism Spectrum Screening Questionnaire (ASSQ)-Revised Extended Version (ASSQ-REV): an instrument for better capturing the autism phenotype in girls—a preliminary study involving 191 clinical cases and community controls. Res Dev Disabil. 2011;32(6):2875-2888. doi:10.1016/j.ridd.2011.05.017 21664105

[25] Kaufman J, Schweder AE. The Schedule for Affective Disorders and Schizophrenia for School-Age Children: Present and Lifetime version (K-SADS-PL). In: Hilsenroth MJ, Segal DL, eds. Comprehensive Handbook of Psychological Assessment, Volume 2: Personality Assessment. John Wiley & Sons; 2004:247-255.

[26] Birmaher B, Brent DA, Chiappetta L, Bridge J, Monga S, Baugher M. Psychometric properties of the Screen for Child Anxiety Related Emotional Disorders (SCARED): a replication study. J Am Acad Child Adolesc Psychiatry. 1999;38(10):1230-1236. doi:10.1097/00004583-199910000-00011 10517055

[27] Swanson JM, Schuck S, Porter MM, . Categorical and dimensional definitions and evaluations of symptoms of ADHD: history of the SNAP and the SWAN rating scales. Int J Educ Psychol Assess. 2012;10(1):51-70. 26504617 PMC4618695

[28] Conners CK, Pitkanen J, Rzepa SR. Conners 3rd Edition (Conners 3; Conners 2008). In: Kreutzer JS, DeLuca J, Caplan B, eds. Encyclopedia of Clinical Neuropsychology. Springer; 2011. doi:10.1007/978-0-387-79948-3_1534.

[29] Bird HR, Shaffer D, Fisher P, Gould MS. The Columbia Impairment Scale (CIS): pilot findings on a measure of global impairment for children and adolescents. Int J Methods Psychiatr Res. 1993;3(3):167-176.

[30] Wagner RK, Torgesen JK, Rashotte CA, Pearson NA. CTOPP-2: Comprehensive Test of Phonological Processing. Pro-Ed; 2013.

[31] Wechsler D. Wechsler Individual Achievement Test. 2nd ed. Psychological Corporation; 2001. doi:10.1037/t15173-000.

[32] Nikolaidis A, Paksarian D, Alexander L, . The Coronavirus Health and Impact Survey (CRISIS) reveals reproducible correlates of pandemic-related mood states across the Atlantic. Sci Rep. 2021;11(1):8139. doi:10.1038/s41598-021-87270-3 33854103 PMC8046981

[33] DeRosa J, Friedman NP, Calhoun V, Banich MT. Neurodevelopmental subtypes of functional brain organization in the ABCD study using a rigorous analytic framework. Neuroimage. 2024;299:120827. doi:10.1016/j.neuroimage.2024.120827 39245397 PMC11779700

[34] Fair DA, Bathula D, Nikolas MA, Nigg JT. Distinct neuropsychological subgroups in typically developing youth inform heterogeneity in children with ADHD. Proc Natl Acad Sci U S A. 2012;109(17):6769-6774. doi:10.1073/pnas.1115365109 22474392 PMC3340031

[35] Tonks J, Yates P, Slater A, Williams WH, Frampton I. Visual-spatial functioning as an early indicator of socioemotional difficulties. Dev Neurorehabil. 2009;12(5):313-319. doi:10.3109/17518420903087913 20477560

[36] Secora K, Emmorey K. Social abilities and visual-spatial perspective-taking skill: deaf signers and hearing nonsigners. J Deaf Stud Deaf Educ. 2019;24(3):201-213. doi:10.1093/deafed/enz006 30882873 PMC6546156

[37] Mix KS, Cheng YL. The relation between space and math: developmental and educational implications. Adv Child Dev Behav. 2012;42:197-243. doi:10.1016/B978-0-12-394388-0.00006-X 22675907

[38] Georges C, Cornu V, Schiltz C. Spatial skills first: the importance of mental rotation for arithmetic skill acquisition. J Numer Cogn. 2019;5(1):5-23. doi:10.5964/jnc.v5i1.165

[39] Kim H, Cameron CE. Implications of visuospatial skills and executive functions for learning mathematics. AERA Open. Published online October 27, 2016. doi:10.1177/2332858416675124

[40] Frick A, Möhring W. A matter of balance: motor control is related to children’s spatial and proportional reasoning skills. Front Psychol. 2016;6:2049. doi:10.3389/fpsyg.2015.02049 26793157 PMC4709580

[41] Clements-Stephens AM, Vasiljevic K, Murray AJ, Shelton AL. The role of potential agents in making spatial perspective taking social. Front Hum Neurosci. 2013;7:497. doi:10.3389/fnhum.2013.00497 24046735 PMC3763481

[42] Bloemen AJP, Oldehinkel AJ, Laceulle OM, Ormel J, Rommelse NNJ, Hartman CA. The association between executive functioning and psychopathology: general or specific? Psychol Med. 2018;48(11):1787-1794. doi:10.1017/S0033291717003269 29521611

[43] Wade M, Zeanah CH, Fox NA, Nelson CA. Global deficits in executive functioning are transdiagnostic mediators between severe childhood neglect and psychopathology in adolescence. Psychol Med. 2020;50(10):1687-1694. doi:10.1017/S0033291719001764 31391139 PMC8026012

[44] Broitman J, Melcher M, Margolis A, Davis JM. Conclusions. In: NVLD and Developmental Visual-Spatial Disorder in Children. Springer; 2020:159-161. doi:10.1007/978-3-030-56108-6_16

