# Supplementary material for: Profiles in Nonverbal Learning Disability, Academic Skills, and Psychiatric Diagnoses in Children
Source: JAMA Netw Open. 2025 Oct 1;8(10):e2533848. doi: 10.1001/jamanetworkopen.2025.33848 (PMC12489661; doi:10.1001/jamanetworkopen.2025.33848)
Supplement: Supplement 1. — eMethods. eTable 1. Subtest Means by Profile eTable 2. DSM-5 Diagnosis Odds Ratios by Profile eTable 3. DSM-5 Diagnoses by Profile eFigure 1. Louvain Bagging Cluster Stability Proportions eFigure 2. Unique Profiles Obtained Across 1000 Bootstrapped Iterations eFigure 3. NVLD Comorbid Diagnosis Prevalence Differences. eFigure 4. Visual-Spatial Reasoning Ability and Domains of Functional Impairment by Louvain Clustering Profiles eReferences. [file jamanetwopen-e2533848-s001.pdf]

Supplemental Online Content

Margolis AE, DeRosa J, Kang M, et al. Profiles in nonverbal learning disability, academic skills, and psychiatric diagnoses in children. *JAMA Netw Open*. 2025;8(9):e2533848. doi:10.1001/jamanetworkopen.2025.33848

eMethods.
eTable 1. Subtest Means by Profile
eTable 2. *DSM-5* Diagnosis Odds Ratios by Profile
eTable 3. *DSM-5* Diagnoses by Profile
eFigure 1. Louvain Bagging Cluster Stability Proportions
eFigure 2. Unique Profiles Obtained Across 1000 Bootstrapped Iterations
eFigure 3. NVLD Comorbid Diagnosis Prevalence Differences.
eFigure 4. Visual-Spatial Reasoning Ability and Domains of Functional Impairment by Louvain Clustering Profiles
eReferences.

This supplemental material has been provided by the authors to give readers additional information about their work.

© 2025 Margolis AE et al. *JAMA Network Open*.

Supplemental Methods

Measures Used in NVLD Criteria

The Wechsler Intelligence Scale for Children-Fifth Edition (WISC-V)[1] is a widely used measure of intelligence measuring 5 aspects of cognition. The Visual Spatial Index is composed of the Block Design and Visual Puzzles subtests and measures visual-spatial processing. Block Design specifically measures visual-spatial reasoning and visual-motor integration. The Fluid Reasoning Index also measures aspects of visual-spatial reasoning including nonverbal inductive and quantitative reasoning and is composed of the Matrix Reasoning and Figure Weights subtests. Matrix Reasoning specifically measures nonverbal, abstract inductive reasoning. The Verbal Comprehension Index is composed of the Vocabulary and Similarities subtests and measures verbal reasoning, understanding, and concept formation and fund of verbal knowledge.

The Wechsler Individual Achievement Test-III (WIAT-III)[2] is a norm referenced academic assessment battery that produces age-adjusted standard scores. The Word Reading subtest assesses the ability to read and decode real words. The Numerical Operations subtest measures knowledge of math computation skills.

The Autism Spectrum Screening Questionnaire (ASSQ)[3] is a parent-completed questionnaire that assesses the presence of 27 Autism Spectrum Disorder symptoms rated on a three-point scale.

The Grooved Pegboard is a norm referenced measure of motor coordination[4]. This task requires the participant to manipulate pegs using their dominant hand and produces a z-score.

The Social Problems Scale from the Child-Behavior Checklist (CBCL)[5] completed by parents measures difficulty with social skills and in peer interactions. The scale yields an age-adjusted T score.

The Flanker and Dimensional Card Sort tests from the NIH Toolbox for Assessment of Neurological Behavioral Function[6] measures attention and inhibitory control as well as cognitive flexibility, respectively. Each yields an age-adjusted standard score.

Measures of Psychiatric and Achievement Symptoms

The Screen for Child Anxiety Related Disorders (SCARED)[7] parent and self-report measures youth anxiety symptoms. The SCARED consists of 41 items and uses a three-point scale (0 = Not True or Hardly Ever True, 1 = Somewhat or Sometimes True, 2 = Very True or Often True). The ratings align to likelihood of different anxiety disorders, specifically, generalized anxiety disorder, panic disorder, separation anxiety, social anxiety disorder, and school avoidance and also yield a Total Anxiety score (range 0-82).

The Strengths and Weaknesses of ADHD Symptoms and Normal Behavior Scale (SWAN),[8] an 18-item parent completed questionnaire assesses symptoms of inattention and hyperactivity/impulsivity.

© 2025 Margolis AE et al. *JAMA Network Open*.

The Conners ADHD Rating Scales Self Report Short Form[9] is a 41-item questionnaire, which was completed by children ages 8-18, produces five scores: Inattention, Hyperactivity, Aggression, Family Relations, and Learning Problems.

The WISC-V Processing Speed Index (PSI) tasks measure the ability to complete paper and pencil tasks within a time limit.[1]

The Rapid Symbolic Naming (RSN) tasks from the Comprehensive Test of Phonological Processing, second edition[10] assess the ability to quickly name symbols. Specifically, during Rapid Digit Naming and Rapid Letter Naming the individual names digits and letters as quickly as possible.

The Reading Comprehension subtest from the WIAT[2] assesses a student's ability to read a short passage and answer questions about what he/she read.

The Columbia Impairment Scale (CIS) includes 13 items rated on a 4-point scale that query about impairment across different life domains e.g., interpersonal relationships, occupational/academic functioning, and use of leisure time.[11] The scale yields a total score with a range from 0-52.

A best estimate clinical diagnosis is made following completion of clinical interviews (Kiddie Schedule of Affective Disorders [KSADS]), the clinically synthesized diagnoses (i.e., consensus DSM-5 diagnoses), and review of all materials collected during study participation.

Louvain Community Detection

To delineate data-driven NVLD profile, we used LCD[12], an iterative network analytic approach that detects sub-groups through two steps. First, the algorithm applies a "greedy" assignment of nodes to communities, favoring local optimizations of modularity. Second, the definition of a new coarse-grained network in terms of the communities found in the first step. Both steps are repeated until no further modularity-increasing reassignments of communities are possible. LCD aims to find a partition of the graph that maximizes modularity (Q). Optimal cluster assignments are algorithmically determined by the set of node assignments that return the highest Q. An attractive feature of LCD for data-driven clustering compared to other such methods (i.e., hierarchical, k-means) is the algorithmic determination of the number of clusters. This feature can help inhibit incorrect cluster approximation and potential researcher bias in cluster choice.

Although LCD was initially developed for use with physical or predefined networks, its application has since expanded to domains where networks of individuals are derived from pairwise similarity across multivariate data, including behavioral, cognitive, and neuroimaging domains.[13] In our study, we transformed the multivariate diagnostic criteria for NVLD into a participant-by-participant similarity matrix by computing Spearman correlations across all features used to define NVLD. Formally, for a dataset $X \in Rn \times p$, where $n=180$ participants and p is the number of diagnostic features, we compute a similarity matrix $S \in Rn \times n$, where each

© 2025 Margolis AE et al. *JAMA Network Open*.

element $Sij=\rho(xi,xj)$, the Spearman correlation between participant i and participant j across all features. This symmetric matrix S becomes the weighted adjacency matrix of an undirected graph G=(V,E), where nodes, V, are individual participants and edge weights, E, represent pairwise similarity.

This procedure does not assume that the data are inherently graph-structured; rather, it mathematically induces a graph from the structure of the data. This network representation is standard in many data-driven subtyping and community detection applications in psychology and psychiatry. Our analytic pipeline mirrors that of DeRosa et al. (2024),[14] who applied LCD to neuroimaging data from the ABCD study. Their work validated the resulting subtypes using bootstrapping, downsampling, and split-sample classification, demonstrating that the similarity-derived graph structure supports stable and interpretable community detection. Nikolaidis et al. (2021)[15] similarly applied LCD to behavioral data and reported stable and meaningful subgroups across different samples and settings.

Louvain automatically determines the number of profiles in a sample by maximizing modularity Q. This metric quantifies how strongly nodes (in this case, participants) are connected within clusters compared to between clusters. Specifically, the algorithm iteratively partitions the network to maximize the modularity function. The number of clusters, or profiles, is determined by the partition that yields the highest Q without the need to predefine how many clusters the model should extract.

LCD offers distinct advantages over distance-based clustering algorithms such as k-means or agglomerative hierarchical clustering. Unlike these approaches, LCD does not require the number of clusters to be specified a priori. It is also robust to noise and sampling variability, and it can identify clusters with irregular or non-linearly separable boundaries. These properties are particularly advantageous when applied to clinical or diagnostic datasets, where the underlying latent subtypes are often heterogeneous, non-Gaussian, and not easily recoverable by centroid- or distance-based methods. This makes LCD especially well-suited for detecting clinically meaningful profiles in populations such as NVLD.

Moreover, the modularity function optimized by LCD: $Q=12m\sum i,j[Aij-kikj2m]\delta(ci,cj)$, is agnostic to the origin of the graph. It requires only a valid weighted adjacency matrix, which our correlation-based similarity matrix provides. As such, LCD is not misapplied in this context; it is operating over a fully valid graph representation of participant similarity, derived from well-defined NVLD-related features.

Measures used in the LCD were assessed for multicollinearity and unidimensionality with exploratory factor analysis and confirmatory factor analysis to ensure proper feature inclusion. All of the variables that were used met the criteria for inclusion in the clustering algorithms.

© 2025 Margolis AE et al. *JAMA Network Open*.

Using Spearman's correlation as the distance metric we first created a *k*-nearest neighbors (knn) graph of each sample. To prevent overfitting and bolster profile robustness and reliability, K for the knn graph was determined using the nclass Sturges formula which implicitly bases k on the range of data.[15] A weighted network is then created by applying a Fisher's exact test to compute the statistical significance of the overlap in neighbors between each pair of samples. As the final step, LCD is applied on the weighted network. Bagging (i.e, bootstrap aggregation) was applied to our LCD analysis, given recent prior work demonstrating the beneficial impact of bagging on cluster reproducibility.[16]

The algorithm takes as input a matrix of N single-cell measurements and partitions them into subpopulations by clustering a graph that represents their phenotypic similarity. It then builds this graph in two steps. First, it finds the *k* nearest neighbors for each cell resulting in N sets of *k*-neighborhoods. Second, it operates on these sets to build a weighted graph such that the weight between nodes scales with the number of neighbors they share. Modularity (Q) of a graph is a quantitative measure of the number of edges found within communities compared against the number predicted in a random graph with an equivalent degree distribution. Positive Q values indicate that the number of intra community edges exceeds those predicted statistically. Q can range from −1.0 to +1.0, with 0 indicating there are no subgroups and 1.0 indicating perfectly reliable division of groups. A wide range of Q may be found for a graph, depending on how nodes are assigned to communities.

Bagging Procedure

Bagging begins by resampling a dataset with replacement (i.e., bootstrapping), and then aggregating across bootstrap samples. The goal of this technique is to reduce variability in the estimation process through averaging across multiple resampled datasets. When applied to clustering, bagging has been shown to improve robustness and reliability (Figure S1, S2). The fundamental advantage of bagging stems from the additional value of combining multiple cluster assignments into a single clustering solution. More specifically, the features for ensemble clustering consist of the aggregation of cluster outputs themselves. LCD is then applied, resulting in cluster solutions for each bootstrap. Each cluster solution is transformed into individual adjacency matrices that are summed together to create an adjacency matrix (similarity matrix) of the total number of times participants were in the same cluster. Mask adjacency matrices are also created and summed to equate the total number of times participants went into the same LCD iteration together (inclusion matrix). The similarity matrix is then divided by the inclusion matrix to create a mean adjacency matrix (stability matrix). That is then turned into a weighted network on which LCD is applied to create the final cluster solution.

Assessing Comorbidities via Chi Square

Chi-square tests of independence evaluated if DSM-based clinical diagnoses (e.g., ADHD) varied across the LCD-derived subtypes. These tests are suitable for categorical outcome data and enabled us to determine if subtype membership was associated with differential prevalence of specific diagnostic categories. For each diagnosis, we computed a 4 × 2 contingency table (4 profiles × presence/absence of diagnosis), calculated observed and expected frequencies under the null hypothesis of independence, and used the standard chi-square formula: $\chi 2=\sum(O-E)2E$. We also verified that all expected cell counts met the minimum criteria. For significant omnibus results, we followed up with pairwise Z-tests for differences in proportions, with Bonferroni correction applied (Figure S3).

© 2025 Margolis AE et al. *JAMA Network Open*.

Associations of Visual-spatial Ability and Functional Domains

Pearson's correlations tested associations between each visual-spatial ability index (FRI, VSI) and one of the four areas of possible impairment (EF, math, fine-motor, and social function), within each profile and across all participants. Each correlation is presented in Figure S4.

© 2025 Margolis AE et al. *JAMA Network Open*.

Table S1. Subtest Means by Profile.

	Profile 1	Profile 2	Profile 3	Profile 4			
	n = 44	n = 37	n = 35	n = 64			
Measure	M (SD)	M (SD)	M (SD)	M (SD)	F	p	Post Hoc
**Similarities**	9.27 (2.54)	9.97 (3.59)	11.54 (3.11)	7.55 (2.86)	14.42	<.001	3 > 1 = 2 > 4
**Vocabulary**	9.27 (2.6)	9.97 (4.12)	11.23 (3.45)	7.67 (2.65)	10.6	<.001	3 > 1 = 2 > 4
**Visual Puzzles**	6.8 (2.21)	6.76 (2.87)	9.6 (2.99)	8.81 (2)	13.94	<.001	3 = 4 > 1 = 2
**Figure Weights**	6.59 (1.9)	8.95 (2.58)	8.11 (2.71)	8.69 (2.29)	8.96	<.001	1 < 2 = 3 = 4
**Coding**	6.34 (2.91)	7.73 (3.49)	6.83 (3.41)	7.33 (2.73)	1.61	>.05	
**Block Design**	5.95 (1.89)	5.43 (2.49)	9.09 (2.06)	7.28 (1.84)	23.59	<.001	3 > 4 > 1 = 2
**Matrix Reasoning**	5.5 (1.89)	8.89 (2.81)	8 (2.41)	8.7 (2.67)	17.88	<.001	1 < 2 = 3 = 4
**Symbol Search**	7.23 (2.28)	8.19 (3.28)	7.29 (2.46)	7.94 (2.47)	1.38	>.05	
**Digit Span**	7.66 (2.05)	8.92 (2.98)	8.71 (3.3)	8.39 (3.02)	1.53	>.05	
**Picture Span**	7.77 (2.55)	9.89 (3.04)	8.4 (3.46)	8.39 (2.78)	3.73	>.05	

© 2025 Margolis AE et al. *JAMA Network Open*.

Table S2. DSM Diagnosis Odds Ratios by Profile

Diagnosis	Profile 1	Profile 2	Profile 3	Profile 4
**Anxiety Disorders**	0.72 [0.43, 1.2], p > .05	2.19 [1.31, 3.66], p=.023*	1.02 [0.61, 1.69], p > .05	0.72 [0.46, 1.14], p > .05
**Attention-Deficit/Hyperactivity Disorder**	1.18 [0.73, 1.9], p > .05	0.76 [0.44, 1.32], p > .05	0.84 [0.51, 1.39], p > .05	1.18 [0.77, 1.82], p > .05
**Autism Spectrum Disorder**	0.7 [0.28, 1.75], p > .05	1.01 [0.4, 2.55], p > .05	0.96 [0.4, 2.3], p > .05	1.33 [0.64, 2.77], p > .05
**Communication Disorder**	1.46 [0.75, 2.88], p > .05	0.94 [0.42, 2.11], p > .05	0.98 [0.47, 2.07], p > .05	0.75 [0.38, 1.47], p > .05
**Depressive Disorders**	1.31 [0.45, 3.8], p > .05	1.37 [0.43, 4.31], p > .05	0.73 [0.21, 2.6], p > .05	0.78 [0.27, 2.27], p > .05
**Disruptive, Impulse Control, and Conduct Disorders**	1.6 [0.7, 3.69], p > .05	0.53 [0.16, 1.8], p > .05	0.99 [0.39, 2.52], p > .05	0.94 [0.41, 2.16], p > .05
**Specific Learning Disorder**	0.71 [0.32, 1.58], p > .05	0.2 [0.05, 0.84], p=.012*	1.64 [0.81, 3.29], p > .05	1.65 [0.87, 3.15], p > .05

Note: Values in brackets indicate 95 percent confidence intervals. *= p<.05 with Bonferroni correction for 7 tests.

© 2025 Margolis AE et al. *JAMA Network Open*.

Table S3. DSM5 Diagnoses by Profile

	Profile 1	Profile 2	Profile 3	Profile 4			
	n = 44	n = 37	n = 35	n = 64			
Diagnosis	N (%)	N (%)	N (%)	N (%)	$\chi 2$	p	Post Hoc
**Anxiety Disorders**	24 (23.76%)	33 (42.86%)	27 (29.03%)	35 (24.48%)	9.67	.022	2 > 1 = 4
**Attention-Deficit/Hyperactivity Disorder**	35 (34.65%)	21 (27.27%)	27 (29.03%)	49 (34.27%)	3.67	>.05	
**Autism Spectrum Disorder**	6 (5.94%)	6 (7.79%)	7 (7.53%)	13 (9.09%)	1.05	>.05	
**Communication Disorder**	14 (13.86%)	8 (10.39%)	10 (10.75%)	13 (9.09%)	1.43	>.05	
**Depressive Disorders**	5 (4.95%)	4 (5.19%)	3 (3.23%)	5 (3.5%)	0.55	>.05	
**Disruptive, Impulse Control and Conduct Disorders**	9 (8.91%)	3 (3.9%)	6 (6.45%)	9 (6.29%)	1.94	>.05	
**Specific Learning Disorder**	8 (7.92%)	2 (2.6%)	13 (13.98%)	19 (13.29%)	10.07	.018	2 < 3 = 4

© 2025 Margolis AE et al. *JAMA Network Open*.

Figure S1. Louvain Bagging Cluster Stability Proportions

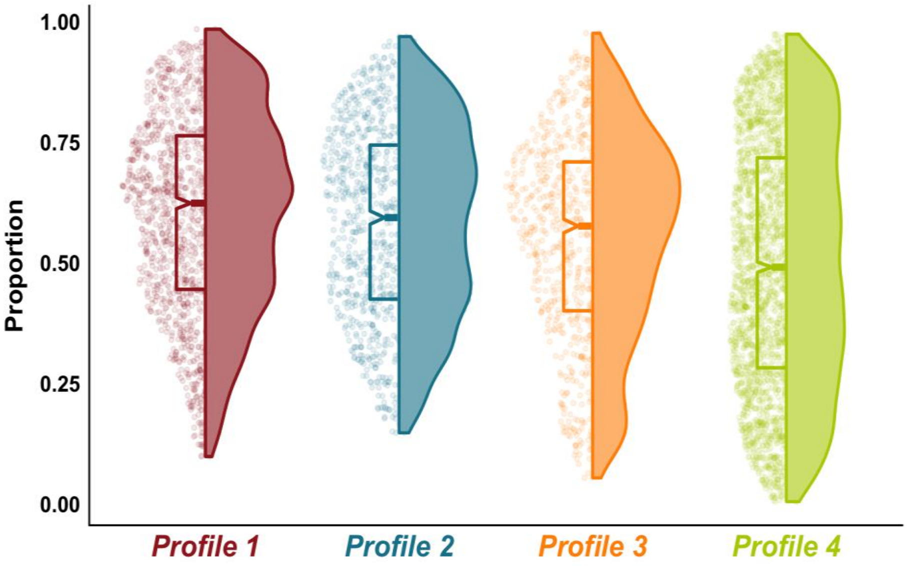

Figure S1. Louvain Bagging Cluster Stability Proportions. Points indicate the proportion of times two given participants were in the same profile across each Louvain iteration they were in. Subject pairwise proportion values were obtained from the bagged stability matrix (See Supplemental *Bagging Procedures* for details regarding how pairwise proportions are obtained).

© 2025 Margolis AE et al. *JAMA Network Open*.

Figure S2. Unique Profiles Obtained Across 1000 Bootstrapped Iterations

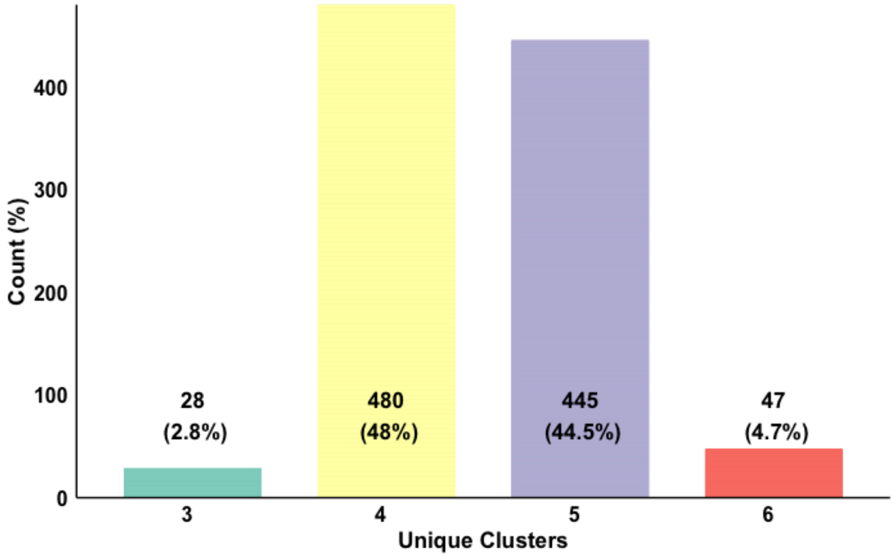

Figure S2. Unique Profiles Obtained Across 1000 Bootstrapped Iterations. Number of unique profile assignments (green, 3; yellow, 4; purple 5; pink 6) that were algorithmically determined by Louvain across 1000 bootstrapped iterations. Values within/above the bars indicate the number of times each assignment appeared and their accompanying proportions in parentheses, respectively.

© 2025 Margolis AE et al. *JAMA Network Open*.

Figure S3. NVLD Comorbid Diagnosis Prevalence Differences.

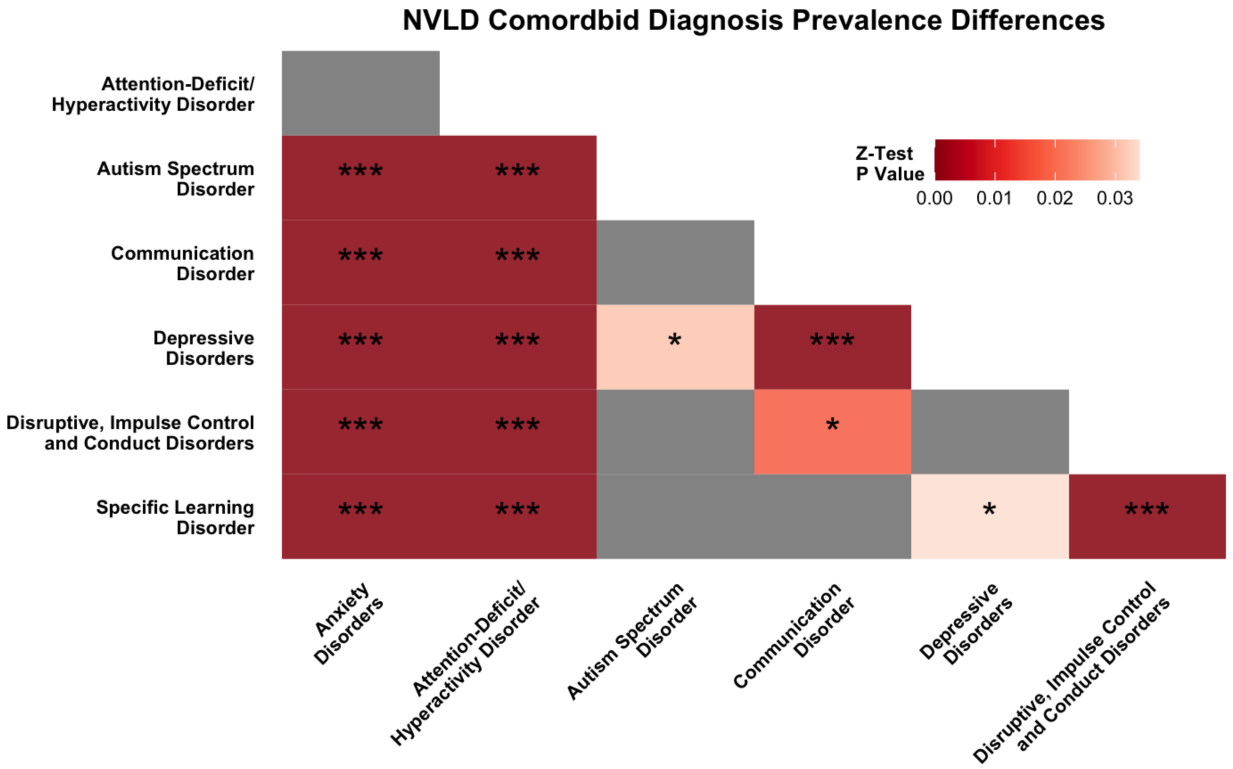

Figure S3. NVLD comorbid diagnosis prevalence differences. Differences were derived using pairwise z-tests. Cell color corresponds to the Z-test p-value level of significance of each diagnostic group pairwise comparison. Asterisks indicate level of significance (*** = p < .001, ** = p <.01, * = p < .05) of the pairwise Z-test comparison. Grey cells indicate p values > .05 and correspond to non-significant diagnostic group prevalence differences.

© 2025 Margolis AE et al. *JAMA Network Open*.

Figure S4. Visual-spatial reasoning ability and domains of functional impairment by Louvain clustering profiles.

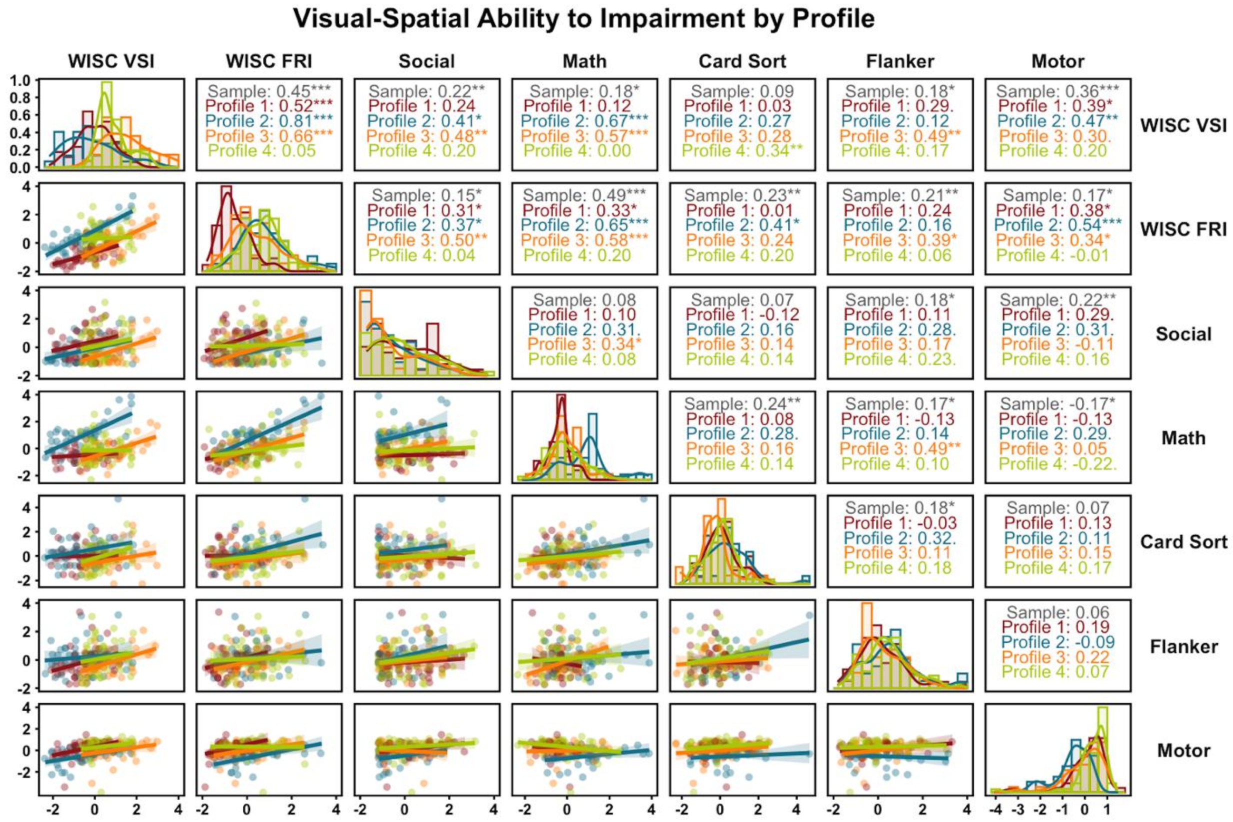

Figure S4. Visual-spatial reasoning ability and domains of functional impairment by Louvain clustering profiles. Scatter plots of each variable pair by profile are presented on the left part of the figure. Pearson correlation for the total sample (all participants) and by profile is displayed on the right. Variable distribution by profile can be found on the diagonal.

© 2025 Margolis AE et al. *JAMA Network Open*.

REFERENCES

1. Wechsler, D. *WISC-V: Technical and Interpretive Manual*. (NCS Pearson, Incorporated, 2014).
2. The Psychological Corporation. *WIAT III: Wechsler Individual Achievement Test, Third Edition: Examiner's Manual*. (2009).
3. Kopp, S. & Gillberg, C. The Autism Spectrum Screening Questionnaire (ASSQ)-Revised Extended Version (ASSQ-REV): An instrument for better capturing the autism phenotype in girls? A preliminary study involving 191 clinical cases and community controls. *Research in Developmental Disabilities* vol. 32 2875–2888 Preprint at https://doi.org/10.1016/j.ridd.2011.05.017 (2011).
4. Strauss, E., Sherman, E. & Spreen, O. *A Compendium of Neuropsychological Texts: Administration, Norms, and Commentary*. (Oxford University Press, Inc, New York, NY, 2006).
5. School-age (CBCL, TRF, YSR, BPM/6-18). https://aseba.org/school-age/ (2019).
6. Zelazo, P. D. *et al.* II. NIH Toolbox Cognition Battery (CB): measuring executive function and attention. *Monogr. Soc. Res. Child Dev.* **78**, 16–33 (2013).
7. Birmaher, B. *et al.* Psychometric properties of the Screen for Child Anxiety Related Emotional Disorders (SCARED): a replication study. *J. Am. Acad. Child Adolesc. Psychiatry* **38**, 1230–1236 (1999).
8. Swanson, J. M. *et al.* Categorical and dimensional definitions and evaluations of symptoms of ADHD: History of the SNAP and the SWAN rating scales. *Int. J. Educ. Psychol. Assess.* **10**, 51–70 (2012).
9. Conners, C. K., Pitkanen, J. & Rzepa, S. R. Conners 3rd Edition (Conners 3; Conners 2008). in *Encyclopedia of Clinical Neuropsychology* (eds. Kreutzer, J. S., DeLuca, J. &

© 2025 Margolis AE et al. *JAMA Network Open*.

Caplan, B.) 675–678 (Springer New York, New York, NY, 2011). doi:10.1007/978-0-387-79948-3_1534.

10. Wagner, R. K., Torgesen, J. K., Rashotte, C. A. & Pearson, N. A. *CTOPP-2: Comprehensive Test of Phonological Processing*. (Pro-Ed, 2013).
11. Bird, H. R., Shaffer, D., Fisher, P. & Gould, M. S. The Columbia Impairment Scale (CIS): Pilot findings on a measure of global impairment for children and adolescents. *Int. J. Methods Psychiatr. Res.* **3**, 167–176 (1993).
12. Blondel, V. D., Guillaume, J.-L., Lambiotte, R. & Lefebvre, E. Fast unfolding of communities in large networks. *Journal of Statistical Mechanics: Theory and Experiment* vol. 2008 P10008 Preprint at https://doi.org/10.1088/1742-5468/2008/10/p10008 (2008).
13. Fair, D. A., Bathula, D., Nikolas, M. A. & Nigg, J. T. Distinct neuropsychological subgroups in typically developing youth inform heterogeneity in children with ADHD. *Proc. Natl. Acad. Sci. U. S. A.* **109**, 6769–6774 (2012).
14. DeRosa, J., Friedman, N. P., Calhoun, V. & Banich, M. T. Neurodevelopmental subtypes of functional brain organization in the ABCD study using a rigorous analytic framework. *bioRxivorg* (2024) doi:10.1101/2024.03.16.585343.
15. Nikolaidis, A. *et al.* The Coronavirus Health and Impact Survey (CRISIS) reveals reproducible correlates of pandemic-related mood states across the Atlantic. *Sci. Rep.* **11**, 8139 (2021).
16. Nikolaidis, A. *et al.* Bagging improves reproducibility of functional parcellation of the human brain. *Neuroimage* **214**, 116678 (2020).

© 2025 Margolis AE et al. *JAMA Network Open*.
