# Supplementary material for: Profiles in Nonverbal Learning Disability, Academic Skills, and Psychiatric Diagnoses in Children
Source: JAMA Netw Open. 2025 Oct 1;8(10):e2533848. doi: 10.1001/jamanetworkopen.2025.33848 (PMC12489661; doi:10.1001/jamanetworkopen.2025.33848)
Supplement: Supplement 2. — Data Sharing Statement [file jamanetwopen-e2533848-s002.pdf]

Data Sharing Statement

Margolis. Profiles in Nonverbal Learning Disability, Academic Skills, and Psychiatric Diagnoses in Children. *JAMA Netw Open*. Published October 01, 2025.
doi:10.1001/jamanetworkopen.2025.33848

Data
Data available: Yes
Data types: Deidentified participant data, Data dictionary
How to access data: https://data.healthybrainnetwork.org/main.php
When available: beginning date: 01-01-2016

Supporting Documents
Document types: None

Additional Information
Who can access the data: Approved reserachers
Types of analyses: For specified purpose
Mechanisms of data availability: Signed DUA
Any additional restrictions: none
